# Annexin A2 Binds RNA and Reduces the Frameshifting Efficiency of Infectious Bronchitis Virus

**DOI:** 10.1371/journal.pone.0024067

**Published:** 2011-08-30

**Authors:** Hoyun Kwak, Min Woo Park, Sunjoo Jeong

**Affiliations:** National Research Lab for RNA Cell Biology, BK21 Graduate Program for RNA Biology, Institute of Nanosensor and Biotechnology, Department of Molecular Biology, Dankook University, Gyeonggi-do, Republic of Korea; BSRC 'Alexander FLEMING', Greece

## Abstract

Annexin A2 (ANXA2) is a protein implicated in diverse cellular functions, including exocytosis, DNA synthesis and cell proliferation. It was recently proposed to be involved in RNA metabolism because it was shown to associate with some cellular mRNA. Here, we identified ANXA2 as a RNA binding protein (RBP) that binds IBV (Infectious Bronchitis Virus) pseudoknot RNA. We first confirmed the binding of ANXA2 to IBV pseudoknot RNA by ultraviolet crosslinking and showed its binding to RNA pseudoknot with ANXA2 protein in vitro and in the cells. Since the RNA pseudoknot located in the frameshifting region of IBV was used as bait for cellular RBPs, we tested whether ANXA2 could regulate the frameshfting of IBV pseudoknot RNA by dual luciferase assay. Overexpression of ANXA2 significantly reduced the frameshifting efficiency from IBV pseudoknot RNA and knockdown of the protein strikingly increased the frameshifting efficiency. The results suggest that ANXA2 is a cellular RBP that can modulate the frameshifting efficiency of viral RNA, enabling it to act as an anti-viral cellular protein, and hinting at roles in RNA metabolism for other cellular mRNAs.

## Introduction

Ribosomal frameshifing is a recoding process of translation where a specific messenger RNA (mRNA)-mediated signal directs a ribosome to shift its reading frame and to continue in the new frame. Programmed-1 ribosomal frameshifting is the most widely used translational recoding mechanism of many viruses [1]. Many viruses employ frameshifting during replication and generate the frameshifted protein critical for efficient viral replication [2], [3], [4]. In the case of retroviruses such as HIV-1, ribosomal frameshifting is required for the expression of protease, reverse transcriptase and integrase. For most other viruses, including coronavirus avian infectious bronchitis virus (IBV), frameshifting generates RNA dependent RNA polymerases. Since translation is a complex process involving many regulatory factors in addition to the ribosome, binding of RNA binding protein (RBP) at a nearby RNA signal could affect RNA conformation and somehow redirect the translational machinery [5], [6]. Therefore, the identification of RBPs involved in frameshifting is important in elucidating the regulatory mechanism for RNA translation.

Viral frameshifting signals are present in coding regions of mRNA, and are generally composed of a “slippery” sequence, a stimulatory downstream RNA hairpin or pseudoknot structure and an intervening spacer region [7], [8], [9]. RNA pseudoknots are structural elements found in almost all classes of RNA, which are now recognized as a widespread motif with diverse biological functions [2]. For example, viral pseudoknots in non-coding regions act in the regulation of translation initiation and in template recognition by viral replicase. In contrast, when pseudoknots are present in coding regions, they regulate translation elongation and termination, leading to ribosomal frameshifting [10]. It is not clear whether ribosomal frameshifting occurs through the interaction of ribosome or any RBPs, or due to the intrinsic nature of the pseudoknot RNA.

Annexin A2 (ANXA2) is a multi-functional protein that has been implicated in a number of cellular functions, including calcium dependent regulation of exocytosis, DNA synthesis and cell proliferation [11]. Recently, its potential role in RNA metabolism was proposed because it was found to bind some mRNA transcripts, such as c-myc, collagen prolyl 4-hydroxylasea (I) and its own mRNA [12], [13], [14], [15], [16]. Since ANXA2 binds 3′-untranslated region (UTR) of these mRNAs, it was suggested to be involved in mRNA localization and translational regulation [15], [17]. Interestingly, ANXA2 is reported to be the most abundant proteins in cytoskeleton bound polyribosome fraction and also associated with mRNAs [17]. These findings led to the suggestion that ANXA2 may bind to some RNA elements involved in translational regulation. In fact, aberrant expression of ANXA2 is somehow related to the carcinogenesis of human cancers, because it has been shown to be clearly absent in some prostate cancers and highly overexpressed in other human cancers [18], [19], [20].

To identify the proteins that might recognize frameshifting signals, we presently utilized IBV pseudoknot RNA as a model transcript. We identified ANXA2 as a RBP that can recognize viral pseudoknot RNA and reduce viral frameshifting. We confirmed the RNA binding activity of ANXA2 by various methods and tested its role in the regulation of frameshifting. By overexpression and knockdown approaches, we showed it to be an efficient modulator of viral frameshifting. We speculate that cellular ANXA2 protein may act as an anti-viral protein that can reduce detrimental viral frameshifting and successful replication of the virus. The present results offer a clue for the role of ANXA2 as a RBP for other cellular RNA transcripts.

## Results

### Identification of Infectious Bronchitis Virus pseudoknot RNA binding proteins

**Figure 1:**
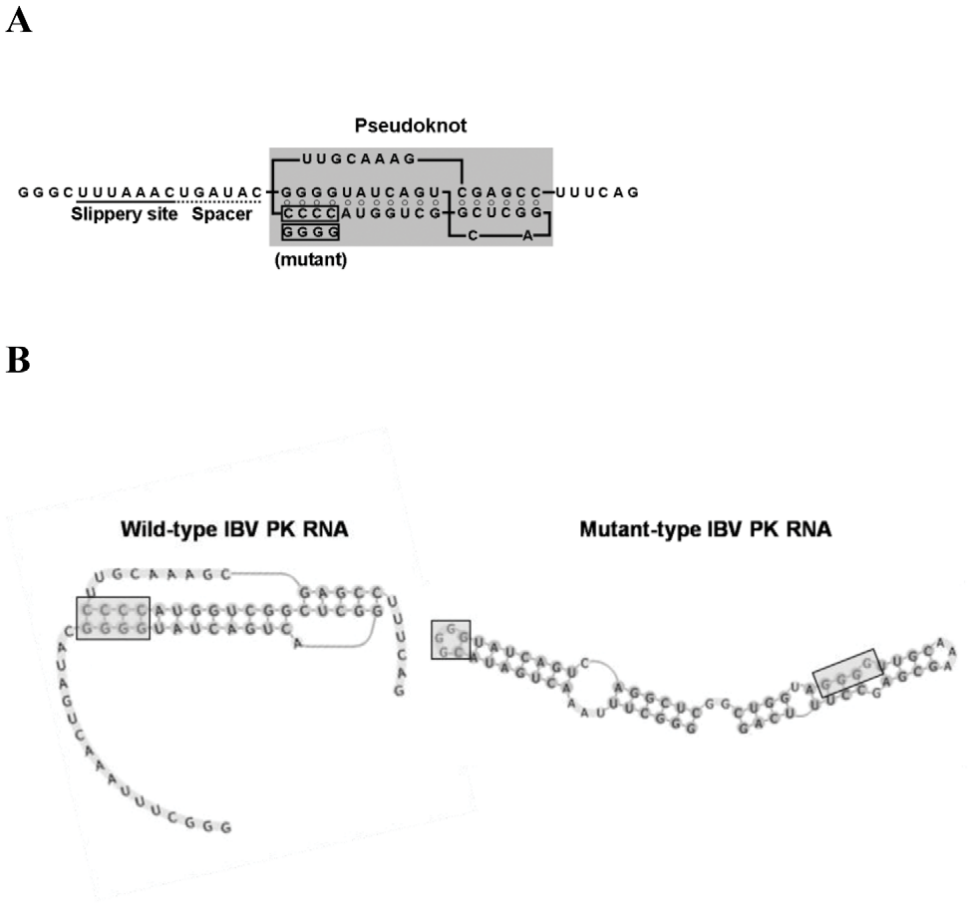
IBV frameshfting elements with slippery site, spacer and pseudoknot. (A) Genomic sequences of IBV frameshifting elements. Mutant refers to the mutation of CCCC to GGGG in the pseudoknot without any change in the slippery site and spacer region. (B) Predicted RNA secondary structure drawn by pknotsRG web program (http://bibiserv.techfak.uni-bielefeld.de/pknotsrg/submission.html) [24], [25]. Wild-type IBV RNA has G-C base pairs in the pseudoknot structure (box), whereas mutant IBV RNA does not form the pseudoknot structure.

Since IBV pseudoknot RNA was shown to induce ribosomal frameshifting during RNA translation, we utilized IBV frameshifting RNA sequences as the model transcript [3]. IBV genomic RNA contains the frameshifting RNA pseudoknot as well as the slippery site and the spacer. Sequences of wild-type and mutant IBV pseudoknot RNA (72 nts) shown in Figure 1A were generated as in vitro transcribed transcript. RNA structure of wild-type IBV RNA was predicted to fold into pseudoknot structure, whereas mutant IBV RNA was not able to form a pseudoknot structure (pknotsRG: http://bibiserv.techfak.uni-bielefeld.de/pknotsrg/submission.html) [21], [22]. These predicted structure are largely consistent with the structure previously reported for IBV pseudoknot RNA by Brierley et al. (1992), albeit with minor differences [9].

**Figure 2:**
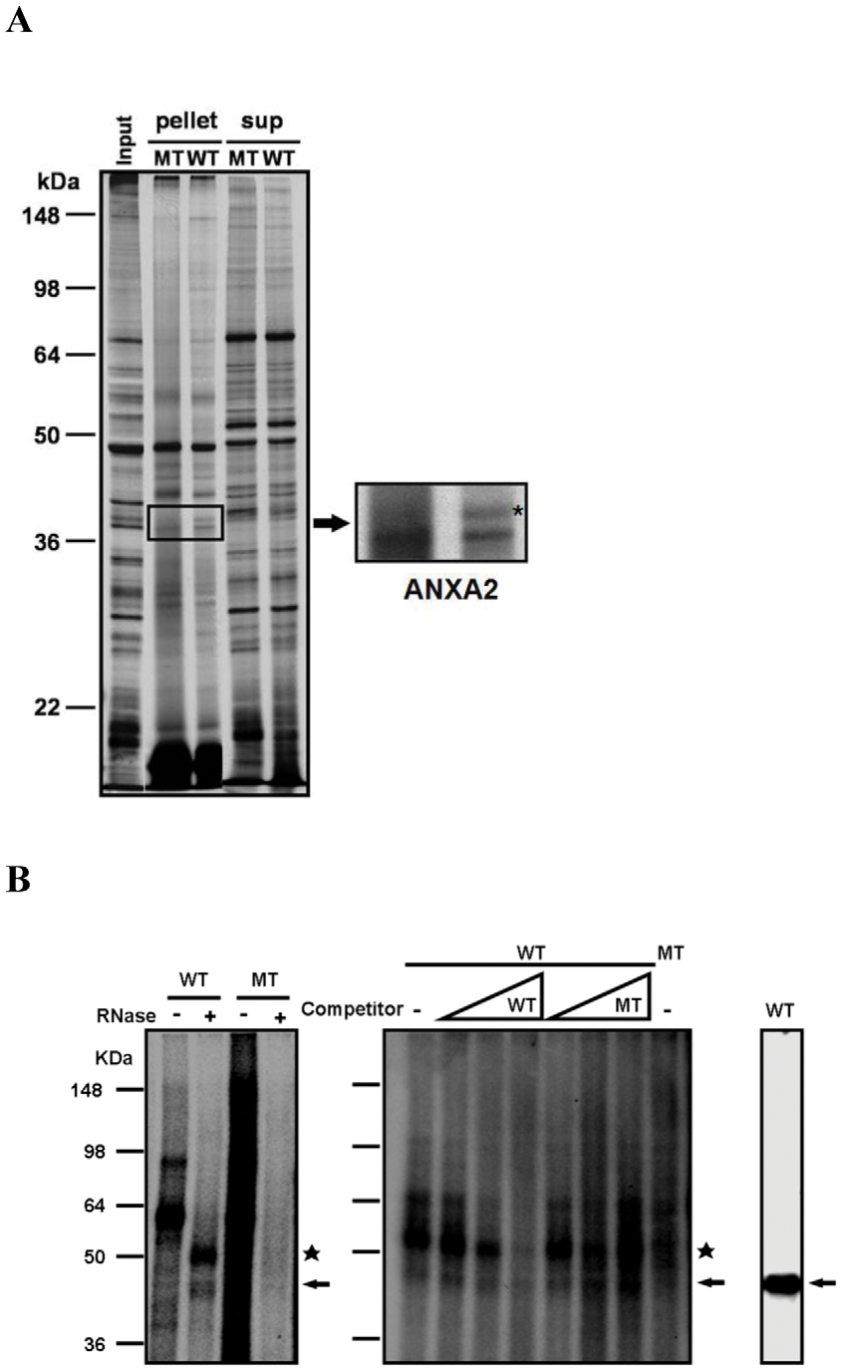
Identification of ANXA2 as the pseudoknot binding protein. (A) Biotin-RNA pull-down assay. Wild-type (WT) or mutant (MT) IBV pseudoknot RNA was in vitro transcribed in the presence of 4-thio UTP with T7 RNA polymerase and 5-end labeled with biotin. Cytosolic extracts were prepared from IMR90 cells, and the extracts were incubated with either wild-type or mutant IBV pseudoknot RNA and precipitated with Streptavidin-coated magnetic beads. Bound proteins (pellet) were eluted from the beads, fractionated using 10% SDS-PAGE and stained with silver. Unbound proteins (sup) were also analyzed as a control. Input refers to 10% input of IMR90 cell extract. Bands isolated from the gel are also shown on the right with higher magnification. MALDI-TOF-TOF analysis identified the wild-type bound protein as ANXA2, indicated with asterisk. (B) UV crosslinking assay (left). IMR90 cell extracts were incubated with the [α-^32^P] labeled Wild-type (WT) or mutant IBV pseudoknot RNA. After crosslinking with UV, RNA-protein complexes were untreated (-) or treated (+) with RNase A and fractionated in polyacrylamide gel. Two protein-RNA complex bands from IMR90 cell extract (shown with an arrow on the right side of the gel, an asterisk indicating non-specific band) were shown to be specific to Wild-type (WT) IBV RNA. Competition assay (middle). Cell extracts were incubated with the [α-^32^P] labeled Wild-type (WT) or mutant (MT) IBV RNA and non-labeled competitor (1×, 10× and 100×, respectively). Western blot analysis of UV-crosslinked RNA-protein complexes (right). The same gel as shown on the left was blotted and incubated with anti-ANXA2 antibody. All data were generated by the results from at least three independent experiments.

To search for cellular proteins that directly interacted with IBV pseudoknot RNA, a RNA pull down assay was performed in the presence of cell extracts (Figure 2A). Wild-type and mutant IBV pseudoknot RNAs were end-labeled with biotin to serve as bait for cellular proteins that might precipitate with streptavidin-coated beads. In addition, RNA was in vitro transcribed in the presence of 4-thio UTP that could serve as crosslinker for directly interacting proteins. IBV pseudoknot RNA was incubated with cell extracts from human lung fibroblast IMR90, which is a natural host cell for the infection of IBV. The complex was irradiated with 365 nm UV light to directly crosslink interacting proteins to 4-thio UTP incorporated IBV pseudoknot RNA. RNA-protein complexes were precipitated with Streptavidin-coated magnetic beads to pull-down any proteins bound to biotin-labeled IBV pseudoknot RNA. Proteins were eluted from the beads, fractionated by SDS-PAGE and analyzed by silver staining. Comparison of the bands from wild-type pseudoknot RNA bound to those from the mutant demonstrated that most of the bound proteins were common for two RNA molecules (Figure 2A). However, a couple of protein bands seemed to present more in wild-type than mutant pseudoknot RNA, so we eluted those bands from the gel. Identification of the protein by MALDI-TOF-TOF revealed that one of such proteins was Annexin A2 (ANXA2).

### ANXA2 specifically binds to pseudoknot RNA

The previous experiment indicated the possible interaction of ANXA2 to IBV pseudoknot RNA. To confirm whether ANXA2 could bind wild-type IBV pseudoknot RNA but not mutant IBV RNA, we performed UV crosslinking assay to show that radiolabeled wild-type IBV pseudoknot RNA bound to ANXA2 protein (Figure 2B). After RNase treatment on RNA-protein complex, radiolabeled IBV pseudoknot RNA bound protein bands were characterized by Western blot analysis with anti-ANXA2 antibody. Radiolabeled RNA was found near the size of the ANXA2 protein, which suggests an interaction between radiolabeled IBV pseudoknot RNA and cellular ANXA2 protein. Significantly, this band specifically competed with wild-type IBV pseudoknot RNA but not with mutant IBV RNA, also supporting the binding specificity of ANXA2 to wild-type pseudoknot RNA (Figure 2B). Identity of the RNA bound protein was also confirmed by Western blot analysis of the same gel (Figure 2B). These data clearly demonstrated binding of ANXA2 to the frameshifting RNA pseudoknot in the IBV genome.

**Figure 3:**
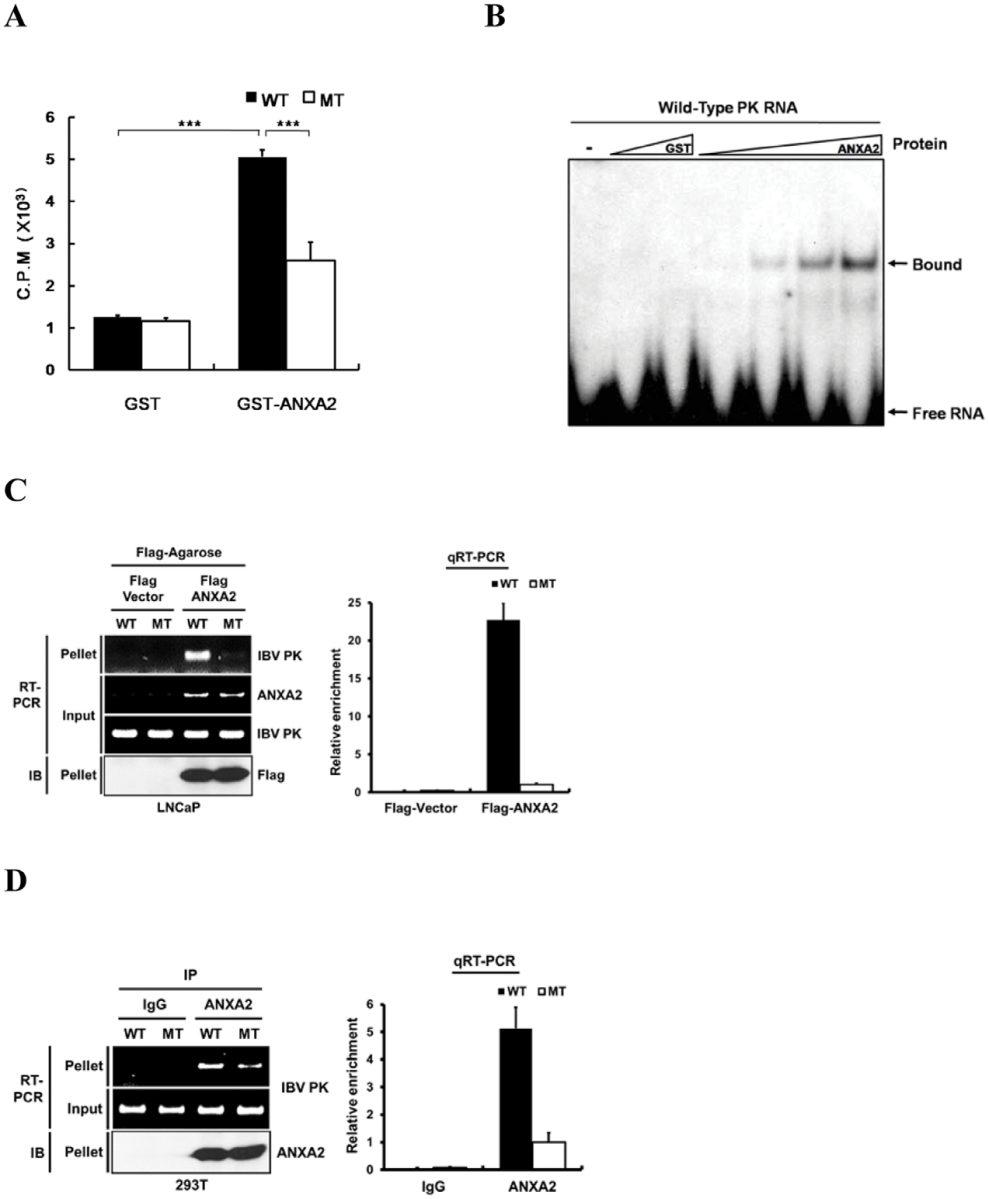
ANXA2 specifically binds to pseudoknot RNA. (A) GST pull-down analysis of IBV RNA. Wild-type (WT) and mutant (MT) IBV pseudoknot RNA were labeled with [γ-^32^P] ATP and mixed with GST or GST-ANXA2 protein. Each sample was pulled down with Glutathione 4B Sepharose and pelleted radioactivity was measured with a scintillation counter. Three independent experiments were performed and statistical analysis was done. All experiments were performed in triplicate and mean ± s.d. are shown. ***: *p<0.001* (B) EMSA with GST or GST-ANXA2 with radiolabeled IBV RNA. Bound and unbound RNA bands are indicated. Lane 1 contains no protein; Lanes 2 and 3 contain GST protein (5 and 10 µM, respectively); Lanes 4-7 contain GST-ANXA2 protein (0.5, 2, 5 and 10 µM, respectively). (C) RNA immunoprecipitation assay. LNCaP cells were co-transfected with reporters (wild-type or mutant IBV pseudoknot plasmid Wild-type or mutant) and ANXA2 expression plasmids (Flag-vector or Flag-ANXA2). After formaldehyde fixation, immunoprecipitations were performed with FLAG-M2 agarose beads. Bound RNA was extracted from the immune complexes and analyzed by RT-PCR (left panel) and qRT-PCR (right panel). PCR products were resolved by electrophoresis in agarose gel and visualized by staining with ethidium bromide. ANXA2 mRNA and IBV PK RNA were also shown as an expression and input controls. Immunoprecipitation of Flag-ANXA2 was confirmed by immunoblotting (IB) using anti-Flag antibody. In the qRT-PCR data, wild-type PK RNA (WT) in immune complex was normalized with input PK RNA level and presented as relative enrichment in comparison to mutant PK RNA (MT). (D) RNA immunoprecipitation assay in HEK293T cells. Reporter plasmids (wild-type or mutant IBV pseudoknot plasmid) were transfected into HEK293T cells and fixed with formaldehyde. Sonicated lysates were then incubated with the antibodies as indicated (anti-IgG or anti-ANXA2). RNA-protein complexes were precipitated with protein-G beads. Bound RNA was extracted from the immune complexes and analyzed by RT-PCR and qRT-PCR. Immunoprecipitated ANXA2 protein was shown by immunoblotting (IB). Relative enrichment of binding was shown as in (C).

Next, we tested whether ANXA2 could bind directly to IBV pseudoknot RNA. To explore this, GST-fused recombinant ANXA2 protein was purified and used for GST pull-down assay. More radioactivity was recovered after precipitating with GST-ANXA2 in comparison to GST, suggesting that recombinant ANXA2 could associate with IBV pseudoknot RNA. Moreover, wild-type IBV pseudoknot RNA tended to bind more than mutant IBV RNA did (Figure 3A). We tested whether such binding was dependent on the presence of calcium, given that ANXA2 is a calcium binding protein. However, calcium did not cause any change in the binding of ANXA2 to IBV pseudoknot RNA (data not shown). We performed a RNA-EMSA to analyze the ANXA2 binding pattern (Figure 3B). RNA-protein complexes were resolved using native polyacrylamide gels. Only a single shifted band was generated in the presence of ANXA2 protein but not with the same amount of the GST protein (Figure 3B). We also confirmed whether ANXA2 bound to wild-type IBV pseudoknot RNA in the cells. Through the RNA-immunoprecipitation assay, we showed that ANXA2 specifically interacted with wild-type IBV pseudoknot RNA but not with mutant IBV RNA in LNCaP and HEK293T cells (Figure 3C and 3D). These data clearly demonstrated the specific binding of ANXA2 to IBV pseudoknot RNA.

### ANXA2 regulates ribosomal frameshifting of IBV pseudoknot RNA

**Figure 4:**
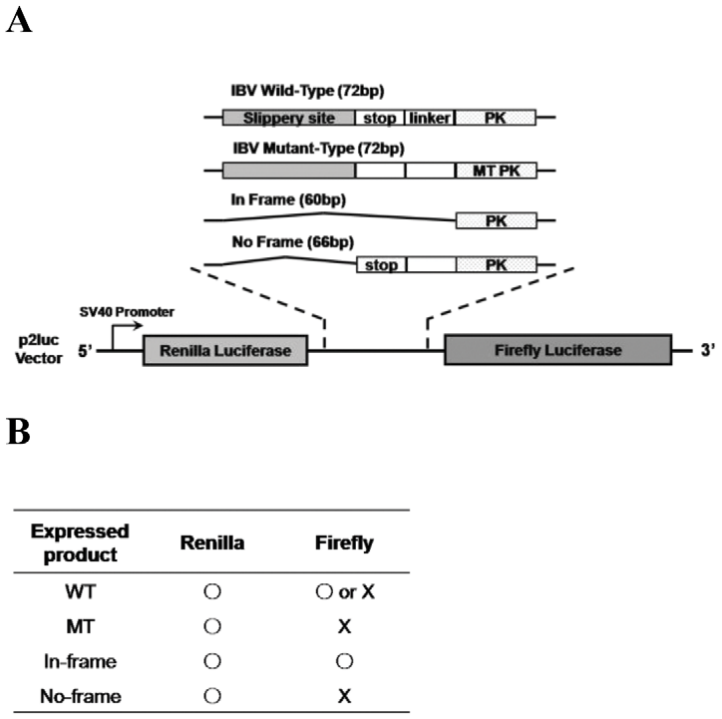
Diagram of frameshifting reporters. (A) Structure of dual luciferase reporter constructs with Renilla and Firefly luciferase genes in p2Luc vector. IBV genomic sequence (72 bp) is composed of slippery site, stop codon, spacer linker and pseudoknot region. In-frame and no-frame control reporters are also shown. Frameshifting efficiency was calculated as described in Materials and Methods. (B) Predicted results from the four reporters.

**Figure 5:**
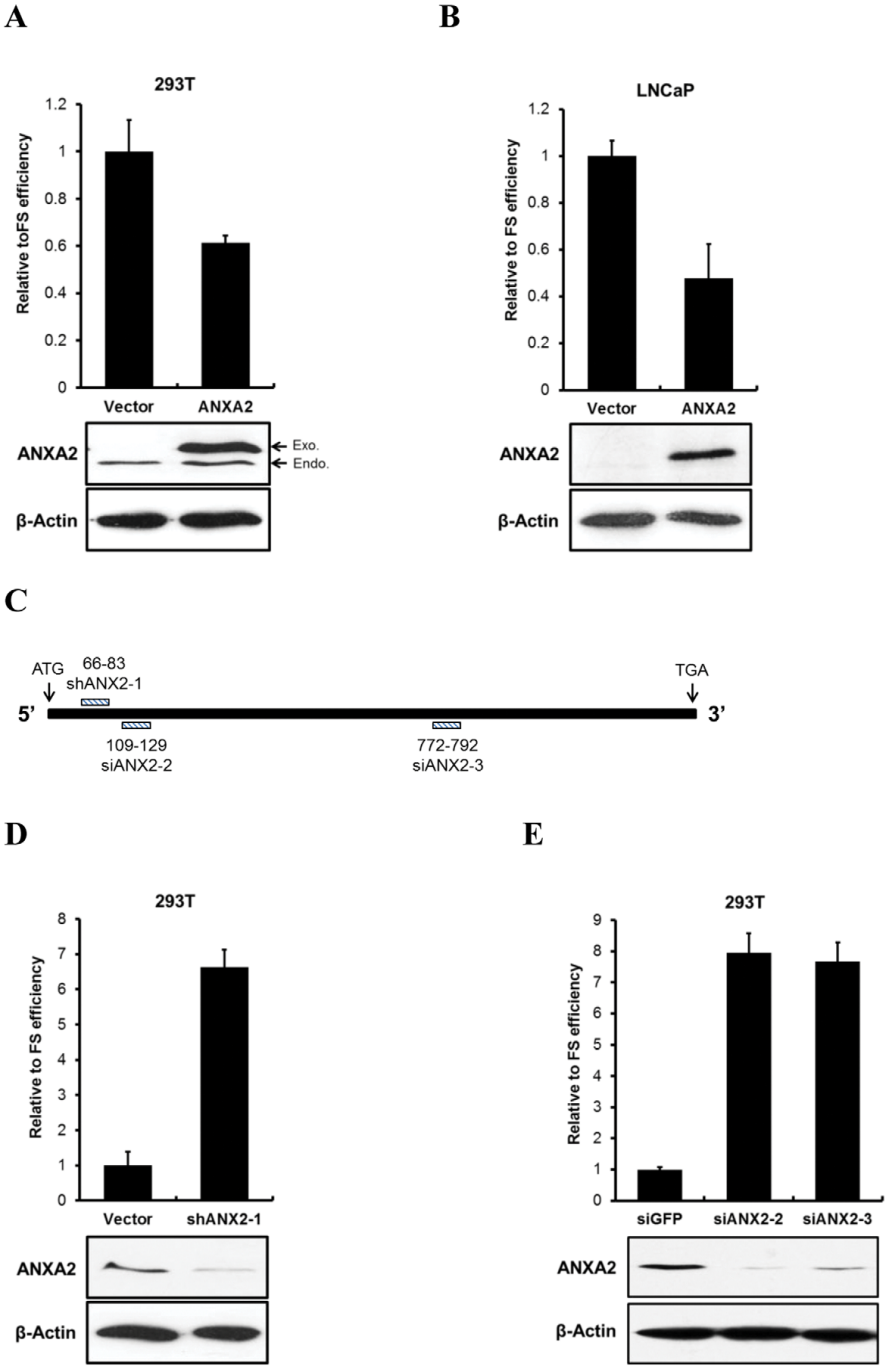
ANXA2 regulates dual luciferase reporters. (A) Dual luciferase reporter assay after overexpression of ANXA2 in HEK293T cells. Relative frameshifting efficiency was measured for the vector and FLAG-tagged ANXA2 transfected cells. Expression of ANXA2 was confirmed by Western blot analysis with anti-ANXA2 antibody. Equal loading of proteins were confirmed by anti-β-actin antibody. (B) Luciferase reporter assay after overexpression of ANXA2 in LNCaP cells. Relative frameshifting efficiency was measured for the vector and FLAG-tagged ANXA2 transfected cells. Expression of ANXA2 was confirmed by Western blot analysis with anti-ANXA2 antibody. Anti-β-actin antibody was used as a loading control. (C) Diagram of siRNA or shRNA target sequences (dashed line) in the coding regions ANXA2 mRNA (filled line). Locations of a shRNA and two siRNAs were indicated (shANX2-1: 66–83 nt, siANX2-2: 109–129 nt, siANX2-3: 772-792 nt). (D) Dual luciferase reporter assay following knockdown of ANXA2 in HEK293T cells. Relative frameshifting efficiency was measured with pSUPER vector (Vector) and with pSUPER-ANXA2 (shANX2-1) transfected cells. Western blot analysis was performed after shRNA transfection. Anti-β-actin antibody was used as a loading control. (E) Luciferase reporter assay after knockdown of ANXA2 in HEK293T cells. Relative frameshifting efficiency was measured from control siRNA (siGFP) or two independent ANXA2 siRNAs (siANX2-2 and -3) transfected cells. Western blot analysis was performed after siRNA transfection. Anti-β-actin antibody was used as a loading control. In (A), (B), (D) and (E), the experiments were performed in triplicate and mean ± s.d. are shown.

Since we found that ANXA2 bound IBV pseudoknot RNA, we next tested whether it could control the efficiency of ribosomal frameshifting on IBV pseudoknot RNA in the cells. We constructed dual luciferase reporters controlled by IBV frameshifting sequences with or without pseudoknot RNA (Figure 4A). Frameshifting of the dual luciferase reporter expressed firefly luciferase as well as Renilla luciferase (Figure 4B), so the frameshifting could be assessed by the ratio between these two proteins as well as by control reporters. Control reporters with either in frame or no expression of firefly luciferase used as positive or negative controls, respectively. To test how ANXA2 regulates the frameshifting efficiency of IBV pseudoknot RNA, we first overexpressed ANXA2 protein in the presence of the reporters and measured the luciferase activities. Transfection of FLAG-tagged ANXA2 in HEK293T cells reduced the relative frameshifting efficiency of IBV pseudoknot RNA up to 40% (Figure 5A). Since HEK293T cells possess an endogenous protein, overexpression of FLAG-ANXA2 might generate additional exogenous ANXA2 protein. To assess more definitively the inhibitory effect of ANXA2 on the frameshifting of IBV pseudoknot RNA, we used a prostate cancer cell line, LNCaP, which does not express endogenous ANXA2 protein in the cells. More significant reduction of the relative frameshifting efficiency was shown in LNCaP cells, which likely resulted from the overexpression of ANXA2 without any basal level expression of the protein (Figure 5B). Conversely, we showed that the downregulation of the ANXA2 by three independent knockdown greatly increased the relative frameshifting efficiency of IBV pseudoknot RNA in HEK293T cells (Figure 5D and 5E). Targeting sequences of shRNA and siRNA in ANXA2 mRNA were diagrammed in Figure 5C. By independent experiments either with shRNA expression vector or with siRNA duplex, we clearly demonstrated that frameshifting efficiency was dramatically increased by ANXA2 knockdown. These data clearly indicated that ANXA2 controls the frameshifting efficiency in the cells, which could act as the cellular inhibitor against the frameshifting of viral RNA translation.

## Discussion

Translation is the complex RNA metabolism regulated by diverse factors, such as ribosome, tRNA and many ribosome associated proteins. Here, we identify ANXA2 as the regulator of ribosomal frameshifting. Since it was previously reported that ANXA2 binds RNA and regulates RNA localization [13], [14], [15], [16], [17], our proteomic identification of ANXA2 as an IBV pseudoknot RNA binding protein leads us to propose that it might regulate the frameshifting efficiency of viral RNA translation. As shown in Figure 5, knockdown of ANXA2 increased the frameshifting efficiency up to seven times, whereas overexpression of ANXA2 significantly down-regulated the frameshifting efficiency of IBV pseudoknot RNA. These results clearly show that ANXA2 reduces IBV viral frameshifting in some way, probably by binding to pseudoknot RNA. Since the frameshifting of IBV generates RNA dependent RNA polymerase, which is critical for successful viral replication, the protein that inhibits frameshifting must be a major antiviral regulator in eukaryotic cells. However, many questions remain to be answered. These include the exact mechanism of ANXA2 action on IBV frameshifting, possible involvement of ANXA2 on frameshifting of other viral RNA and unexplored functions of RNA metabolism on many other pseudoknot containing cellular RNA.

RNA pseudoknots are structural elements formed upon base-pairing of a single-stranded region to a stretch of complementary nucleotides elsewhere in the RNA chain. Since the first discovery of pseudoknot structure in 3′-UTR of Turnip Yellow Mosaic Virus (TYMV), numerous viruses have been reported to contain pseudoknot RNA [7], [8], [9]. Many other cellular RNAs, such as rRNA, mRNA, tmRNA, catalytic RNA, telomerase RNA, RNA components of ribonucleoprotein complex and many artificially selected RNA aptamers also form pseudoknot structures [23], [24], [25], [26], [27]. A pseudoknot is not only a structural element of RNA, but also a functional regulator of RNA. As mentioned previously, RNA pseudoknot located in the coding region of virus is involved in the -1 ribosomal frameshifting of viral RNA translation, and pseudoknot in 5′-UTR in some cases regulates the initiation of viral RNA replication [28]. Since mutational analysis revealed drastic changes of ribosomal frameshifting efficiency upon disruption of pseudoknot structure, this structure is likely to be kept intact in addition to the slippery site and spacer region for efficient frameshifting [29]. Recent advances in RNA bioinformatics broaden the possibility of finding pseudoknot structure from RNA sequences and predicting three-dimensional structures of the RNA molecules [30], [31], [32].

The detailed mechanism of pseudoknot function on frameshifting is being intensively studied. Recent data suggests that the mechanical strength of the pseudoknot structure is correlated with ribosomal frameshifting, with pausing of ribosome itself is not sufficient for frameshifting [33], [34], [35], [36]. While still contentious, several reports describe pseudoknot binding protein factors. For example, 102-kDa RNA binding protein and eukaryotic elongation factor 1 (eEF1) bind RNA pseudoknot of 3′-UTR in the Tobacco Mosaic Virus genome [37], [38]. Recently, it was also reported that replicase gene product of mouse coronavirus interacts with its own RNA pseudoknot [29]. Also, we present data showing that ANXA2 binds pseudoknot RNA and inhibits ribosomal frameshifting of IBV. Even though it is necessary to determine the exact mode of action for ANXA2 on ribosomal frameshifting, evidence is accumulating concerning the role of ANXA2 in translational regulation. For example, ANXA2 is associated with active and inactive mRNAs, polyribosomes and many ribosomal subunit proteins [12], [19]. In addition, the receptor for activated C-kinase (RACK1), which resides in ribosome acting as the signal regulator of translation, also interacts with ANXA2 [39].

Deregulated expression of ANXA2 is implicated in many human tumors and diseases [20], [40]. Especially, ANXA2 has an important role in angiogenesis and tumor progression, and, thus, might be a potential therapeutic target [41]. Furthermore, the gene linked to Optiz BBB/G syndrome, MID1, is associated with microtubule-associated ribonucleoprotein complex including EF-1a, RACK-1, small ribosomal subunit proteins and ANXA2, which again suggests a possible role of ANXA2 in RNA translation and in pathogenesis of the cell [42]. Since viral frameshifting is critical for many pathogenic viruses including coronavirus, ANXA2-pseudoknot RNA interaction might be a novel target for anti-viral drug discovery [43], [44].

## Materials and Methods

### Plasmids

Plasmids containing wild-type (pFS-cass5) and mutant (pFS-cass5.15) pseudoknot sequences from IBV genomic RNA were generous gifts form Dr. Ian Brierley (University of Cambridge) [3]. Bovine ANXA2 cDNA was kindly provided by Dr. Anni Vedeler (University of Bergen) [12].

### Cell culture

Human embryonic kidney cell line HEK293T were purchased from the American Type Culture Collection (ATCC) and maintained in Dulbecco's modified Eagle's medium (DMEM) containing 10% fetal bovine serum (FBS; BRL Life Technology). Human prostate cancer cell line LNCaP were purchased from the ATCC and cultured in RPMI1640 with 10% FBS. Human lung fibroblast IMR90 and human lung adenocarcinoma SK-Lu I cells were purchased from ATCC and cultured in MEM containing 10% FBS using standard techniques.

### Preparation of cytosolic extracts

Cells were harvested and washed twice with ice-cold phosphate buffered saline (PBS). Cell pellets were resuspended in two volumes of buffer A (20 mM HEPES, pH 7.9; 10 mM NaCl; 1 mM EDTA; 1 mM DTT) supplemented with protein inhibitor cocktail (1 mM PMSF, Sigma-Aldrich). After incubating 15 min on ice, cells were lysed with 0.5% Nonidet P-40 and centrifuged at 4,000 rpm at 4°C for 30 sec. Clarified cytosolic extract was quantitated for protein concentration and stored in aliquots at −80°C.

### Biotin-RNA pull down assay

Wild-type and mutant IBV RNAs were transcribed in the presence of 4-thio UTP (Ambion) by in vitro transcription with T7 RNA polymerase. Phosphate was removed from 5′-end by Calf Intestinal Alkaline Phosphatase (New England Bio Lab) and γ-S^32^ was added by incubating with γ-S^32^ ATP (10 mM) with T4 Polynucleotide Kinase (New England Bio Lab). Biotin was added at the 5′-end of the prepared RNA by incubating one and half hours with 10 mM PEO-Iodoacetyl Biotin (PIERCE) in the dark. Cytosolic extracts were pre-cleared with Streptavidin-coated magnetic beads (New England Bio Lab) and beads were pre-blocked with tRNA. Biotin and 4-thio labeled RNA was incubated with pre-cleared cell extract in binding buffer (20 mM HEPES, pH 7.5; 50 mM KCl; 1 mM DTT; 0.1 mM EDTA; 5% glycerol; 40 U RNase inhibitor) for one hour in room temperature. Ultraviolet radiation (365 nm) was performed to induce binding between 4-thio UTP and interacting proteins. Pre-blocked magnetic beads were added and incubated for one hour to pellet RNA-protein complexes. Two volumes of washing buffer (20 mM HEPES, pH 7.5; 500 mM NaCl, 1 mM DTT, 0.1 mM EDTA, 5% glycerol) was added and washed seven times, followed by elution with boiling in 2× sample buffer for 5 min. Proteins were separated by 10% SDS-PAGE and visualized by silver staining .

### Identification of proteins

After pulling-down interacting proteins by a biotin pull-down assay as described below, protein identification was performed in the Functional Proteomics Center, Korea Institute of Science and Technology. Silver stained bands were excised and an automated in-gel tryptic digestion was performed on a Mass Prep Station (Micromass). The gel pieces were destained, reduced with DTT, alkylated with iodoacetamide and digested with sequencing grade modified trypsin (Promega). Resulting peptides were extracted from the gel and analyzed via MALDI-TOF and MALDI-TOF-TOF. Proteins were identified from the mass spectrometry data using MASCOT (Matrix Science) and searching the NCBI data base.

### Ultraviolet (UV) cross-linking assay

IBV pseudoknot RNA was transcribed and labeled in vitro with T7 RNA polymerase (Ambion) with α-[^32^P]CTP. ^32^P-labeled IBV pseudoknot RNA (1.1×10^5^ c.p.m) was incubated with 30 µg of cell extract with 2 µg of tRNA, 0.2 µg of poly d(I-C) and 40 U of RNase inhibitor. The RNA-protein binding reaction was carried out in a 30 µl reaction mixture containing 20 mM HEPES pH 7.5, 50 mM KCl, 1 mM DTT, 0.1 mM EDTA and 5% glycerol. The mixtures were incubated at room temperature for 30 min, after which they were UV-irradiated (120 mJ/cm^2^) on ice for 30 min with an UV cross linker (Fisher Scientific). RNAs were digested with 1 µg RNase A, 25 U RNase S1 and 0.1 U RNase V1 at 37°C for 10 min and analyzed by 10% SDS-PAGE.

### Western blot analysis

Anti-ANXA2 antibody (BD Biosciences) was used for Western blot analysis. Proteins in sample buffer were resolved by 10% SDS-PAGE, transferred to a polyvinylidene difluoride membrane, immunostained by specific antibodies and visualized using a chemiluminescent substrate.

### Glutathione-S-transferase (GST) pull-down assay

GST-pull down assay was performed for confirmation of direct RNA-protein interaction. Radiolabeled RNA transcript (5×10^4^ c.p.m) and purified proteins were incubated at room temperature for 1 h in a binding buffer containing 25 mM HEPES pH 7.5, 100 mM NaCl and 1 mM MgCl_2_. RNA-protein complexes were pulled down by Glutathione Sepharose 4B (GE Healthcare) pre-blocked with tRNA for 30 min. Following washing using binding buffer, the radioactivity in the recovered pellets was measured by scintillation counting.

### Construction of ANXA2 expression clone and RNA interference

cDNA for ANXA2 was polymerase chain reaction (PCR)-amplified and ligated into pCMV-Tag2B vector (stratagene). ANXA2 was amplified with primers 5′ -CAGGATCCATGTCTACCGTTCA-3′ and 5′ -CCGAATTCTCAGTCATCCCCAC-3′. To knock-down ANXA2 mRNA, small hairpin RNA (shRNA) was designed using the Ambion small interfering RNA (siRNA) converter website. The ANXA2 target sequence was 5′-UGCAUAUGGGUCUGUCAA-3′ corresponded to coding region nucleotides 66-83. To make pSUPER–ANXA2 (shANX2-1), specific oligonucleotides were synthesized (Bioneer, Korea) and ligated to pSUPER vector as previously reported [45]. Also, two different siRNAs corresponding to coding region nucleotides 109–129 (siANX2-2: 5′-CGGGAUGCUUUGAACAUUGAA-3′) and 772–792 (siANX2-3: 5′-AACCUGGUUCAGUGCAUUCAG-3′) were designed. siRNA duplex were chemically synthesized and contain dTdT 3′ overhangs (Bioneer, Korea).

### Purification of recombinant ANXA2 protein

cDNA of the ANXA2 protein was PCR amplified and inserted into the pGEX-4T-1 vector (GE Healthcare), followed by the transformation of the vectors into the protease deficient strain BL21. GST-ANXA2 and GST protein was purified according to manufacturer's instructions.

### Electrophoretic Mobility Shift Assay (EMSA)

The basic procedure for EMSA was previously described [46]. Briefly, RNA was in vitro transcribed and labeled in vitro with T7 RNA polymerase (Ambion) with [α-^32^P]ATP. ^32^P labeled IBV pseudoknot RNA (1.1×10^5^ c.p.m) and purified GST-ANXA2 or GST protein were incubated at room temperature for 30 min in a binding buffer containing 25 mM HEPES pH 7.5, 100 mM NaCl, 1 mM MgCl_2_ and 4% glycerol. RNA-protein complexes were resolved on 5% native polyacrylamide gels. Gels were dried and analyzed by autoradiography.

### RNA immunoprecipitation assay (RNA-IP) and qRT-PCR

The basic procedure for RNA-IP was previously described [46]. Briefly, LNCaP and HEK293T cells were transiently co-transfected with wild-type of mutant IBV pseudoknot reporters and pCMV Tag2B vector (Flag-Vector) or FLAG-ANXA2 plasmids, and incubated with 1% formaldehyde for crosslinking. Sonicated lysates were immunoprecipitated with FLAG M2 agarose beads (Sigma Aldrich) or with antibodies as indicated (anti-normal mouse IgG or anti-ANXA2). Pellets were subsequently incubated at 70°C for 1 h to reverse the crosslinks, and the RNA was purified with TRI Reagent (Ambion) and treated with DNase I (Ambion) to remove reporter plasmid DNA. After reverse transcription, cDNA was amplified by using primer pairs for IBV pseudoknot RNA (5′-GTCGACTTTAAACTGATACGGGGTATC-3′ and 5′-GAAGGATCCCAGCTGAAAGGC-3′). qRT-PCR was performed with StepOne real-time PCR system (Applied Biosystems). Reactions were amplified using selective primers as described above with Power SYBR master mix (Applied Biosystems) according to the manufacturer's instructions. Quantification was carried out with the StepOne™ software (Ver 2.2). Percentages of PK RNA binding to ANXA2 were expressed as the ratio of comparative threshold cycle (*C_T_*) to input level in PK mRNA

### Construction of dual luciferase reporters

To make IBV dual luciferase reporters, a frameshifting sequence (72 bp) was amplified from pFS-cass 5 and pFS-cass 5.15. The sequence was inserted between Renilla and firefly luciferase genes of a dual reporter, p2Luc vector (kindly provided by Dr. Yang Kyun Kim, SungKyunKwan University) [47]. Wild-type reporter is composed of slippery site, stop codon, spacer linker and pseudoknot region of IBV genomic sequences. The mutant reporter is as same as the wild-type reporter except for a disrupted pseudoknot. The in-frame reporter has wild-type pseudoknot but not frameshifting regulatory elements, where no-frame reporter has wild-type frameshifting regulatory elements except for the slippery site. Wild-type and mutant IBV pseudoknot sequences were amplified by PCR with primers 5′-AATGTCGACTTTAAACTGATACGGG-3′ and 5′-TAAGGATCCCAGCTGAAAGGCTC- 3′ from of pFS-cass 5 and pFS-cass 5.15, respectively. In frame sequence was amplified with primers 5′-AATGTCGACACGGGGTATCAGTC-3′ and 5′-TAAGGATCCCAGCTGAAAGGCTC-3′. No frame sequence was amplified with primers 5′-AATGTCGACCTGATACGGGGTAT-3′ and 5′-TAAGGATCCCAGCTGAAAGGCTC-3′.

### Dual luciferase assay

Cells were cultured and transfected with frameshifting reporters using Lipofectamine™ (Invitrogen). For the luciferase assay, cells were scraped into 100 µl of passive lysis buffer (Promega). Luciferase activity in the lysate was determined with a dual-luciferase reporter assay system (Promega), according to the manufacturer's instruction and measured with a Turner Luminometer TD-20/20. Firefly luciferase activity was normalized to the activity of renilla luciferase. Frameshifiting efficiency was calculated by the following formula:
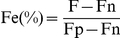
where *Fe*  =  Frameshifting efficiency, *F* =  Wild-type or mutant Luc activity, *Fn* =  No-frame Luc activity and *Fp*  =  In-frame Luc activity.
